# Extending Cryo-EM to Nonaqueous Liquid Systems

**DOI:** 10.1021/acs.accounts.1c00077

**Published:** 2021-04-19

**Authors:** Asia Matatyaho Ya’akobi, Yeshayahu Talmon

**Affiliations:** Department of Chemical Engineering and the Russell Berrie Nanotechnology Institute (RBNI), Technion—Israel Institute of Technology, Haifa 3200003, Israel

## Abstract

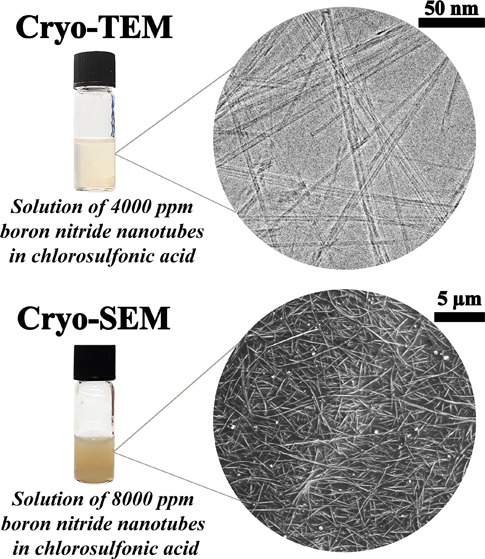

Cryogenic-temperature transmission electron microscopy (cryo-TEM) of aqueous systems has become a widely used methodology, especially in the study of biological systems and synthetic aqueous systems, such as amphiphile and polymer solutions. Cryogenic-temperature scanning electron microscopy (cryo-SEM), while not as widely used as cryo-TEM, is also found in many laboratories of basic and applied research. The application of these methodologies, referred to collectively as cryogenic-temperature electron microscopy (cryo-EM) for direct nanostructural studies of nonaqueous liquid systems is much more limited, although such systems are important in basic research and are found in a very large spectrum of commercial applications. The study of nonaqueous liquid systems by cryo-EM poses many technical challenges. Specimen preparation under controlled conditions of air saturation around the specimen cannot be performed by the currently available commercial system, and the most effective cryogen, freezing ethane, cannot be used for most such liquid systems. Imaging is often complicated by low micrograph contrast and high sensitivity of the specimens to the electron beam.

At the beginning of this Account, we describe the basic principles of cryo-EM, emphasizing factors that are essential for successful direct imaging by cryo-TEM and cryo-SEM. We discuss the peculiarities of nonaqueous liquid nanostructured systems when studied with these methodologies and how the technical difficulties in imaging nonaqueous systems, from oil-based to strong acid-based liquids, have been overcome, and the applicability of cryo-TEM and cryo-SEM has been expanded in recent years. Modern cryo-EM has been advanced by a number of instrumental developments, which we describe. In the TEM, these include improved electron field emission guns (FEGs) and microscope optics, the Volta phase plate to enhance image contrast by converting phase differences to amplitude differences without the loss of resolution by an objective lens strong underfocus, and highly sensitive image cameras that allow the recording of TEM images with minimal electron exposure. In the SEM, we take advantage of improved FEGs that allow imaging at a low (around 1 kV) electron acceleration voltage that is essential for high-resolution imaging and for avoiding specimen charging of uncoated nonconductive specimens, better optics, and a variety of sensitive detectors that have considerably improved resolution and, under the proper conditions, give excellent contrast even between elements quite close on the periodic table of the elements, such as the most important oxygen and carbon atoms.

Finally we present and analyze several examples from our recent studies, which illustrate the issues presented above, including the remarkable progress made in recent years in this field and the strength and applicability of cryo-EM methodologies.

## Key References

- BellareJ. R.; DavisH. T.; ScrivenL. E.; TalmonY.Controlled Environment Vitrification System : An Improved Sample Preparation Technique. J. Electron Microsc. Technol.1988, 111, 87–11110.1002/jemt.10601001113193246.^1^*Description of the controlled environment vitrification system (CEVS), which allows cryogenic electron microscopy specimen preparation under controlled conditions of temperature and air saturation (or dryness, as needed) around the specimen until its vitrification*.
- IssmanL.; TalmonY.Cryo-SEM Specimen Preparation under Controlled Temperature and Concentration Conditions. J. Microsc.2012, 246, 60–6910.1111/j.1365-2818.2011.03587.x22268668.^2^*Extension of the CEVS concept to the preparation of cryogenic temperature scanning electron microscopy specimens under controlled conditions.*
- KleinermanO.; Parra-VasquezA. N. G.; GreenM. J.; BehabtuN.; SchmidtJ.; KesselmanE.; YoungC. C.; CohenY.; PasqualiM.; TalmonY.Cryogenic-Temperature Electron Microscopy Direct Imaging of Carbon Nanotubes and Graphene Solutions in Superacids. J. Microsc.2015, 259, 16–2510.1111/jmi.1224325818279.^3^*Application of cryo-EM at the extreme conditions of solution in “super acids” such as chlorosulfonic acid*.^3^
- LibermanL.; KleinermanO.; DavidovichI.; TalmonY.Micrograph Contrast in Low-Voltage SEM and Cryo-SEM. Ultramicroscopy2020, 218, 11308510.1016/j.ultramic.2020.11308532771863.^4^*Description of several aspects of scanning electron microscopy at low acceleration voltage, including avoiding specimen charging, a change in the micrograph contrast with acceleration voltage, and obtaining good contrast between domains of oil and water in cryo-SEM*.

## Introduction

Cryogenic transmission electron microscopy (cryo-TEM) has become an indispensable tool in the study of a wide range of liquid and semiliquid material systems, both biological and synthetic. The first impetus for cryo-TEM came from biologists who wanted to make their liquid and semiliquid systems compatible with the high vacuum of the electron microscope and arrest all supramolecular level in them without the destructive effect of staining and drying, the common way that had been used for preparing such systems for electron microscopy (EM).^5^ An important breakthrough in the development of the methodology was the work of Dubochet, who demonstrated how to vitrify aqueous systems, thus avoiding crystallization damage to the specimens.^6^ Around the same time, efforts have been made to develop the methodology of cryogenic scanning electron microscopy (cryo-SEM) by pioneers such as Echlin^7^ and Walther and Pawley.^8^ Later on the issue of environmental control around the specimen was addressed by Talmon and co-workers.^1^ That has allowed control over the temperature and concentration of the system during specimen preparation, ensuring that the cryo-EM specimen reflects the state of the original material system. Quite early on it became apparent that cryo-EM, and more specifically, the presence of the vitrified liquid in the specimen, poses more severe electron-beam radiation-damage problems than in dry room-temperature specimens.^9^

Because water is the material of life as we know it and water is also found in most of the everyday synthetically prepared liquids and gels, almost all cryo-EM work has been performed on aqueous systems. The reader is referred to some key interesting and useful publications and reviews on cryo-EM of aqueous liquid systems.^10−14^ However, there are numerous nonaqueous nanostructured liquid and semiliquid systems that could benefit from their study by cryo-EM, but those systems are usually quite more complicated to handle due to issues connected to their preparation, thermal fixation, and imaging. Over the years, members of the cryo-EM community have modified cryo-EM methodologies to allow imaging of nonaqueous systems by cryo-TEM and cryo-SEM.^15−17^ That includes oil-in-water, water-in-oil, and bicontinuous microemulsions; in the latter, both oil and water are continuous.^18,19^ In some cases of practical importance the solvents are strong acids. Even in those extreme cases it has been possible to perform cryo-TEM and cryo-SEM, after the physics and chemistry of these systems during specimen preparation, vitrification and imaging have been studied and the methodologies have been modified to accommodate the special nature of those material systems. However, very little research has been conducted by cryo-EM of nonaqueous liquid systems, partly because the commercial systems for cryo-EM specimen preparation can handle only aqueous systems.

This Account gives a detailed account of the present-day state of cryo-EM of nonaqueous systems, emphasizing the difficulties that arise from the physics and chemistry of specimen preparation, the interaction of the electron beam with the specimen in general and imaging in particular, and the technical solutions used to overcome those difficulties. This is a distillation of the experience that has been collected by the second author’s research group in many years of imaging a very wide range of liquid systems.

## Specimen Preparation

### Thermal Fixation

Cryo-EM involves thermal fixation (i.e., rapid cooling of the specimen at cooling rates that lead to vitrification of the specimen, thus avoiding crystallization of the liquid upon cooling). Crystallization causes the segregation of solutes and leads in the TEM to electron optical artifacts from defects in the ice crystals formed in the specimen. Cryo-TEM specimens are typically thinner than 300 nm, thus they have a very large surface area to volume ratio that is conducive to high rates of heat transfer. Cryo-SEM specimens are much thicker (in the millimeter range), thus other measures must be taken, such as high-pressure freezing (see below).

In the current technology, fast cooling is usually achieved by plunging the specimen into a suitable cryogen, namely, a liquid at very low temperature. In such a process, heat transfer is proportional to the temperature difference between the cooled object and the liquid and to a heat-transfer coefficient (HTC), which reflects the mechanism of the given process. Plunging a specimen into a liquid at a temperature way below its boiling point causes heat transfer by conduction, which is quite effective, leading to a relatively high HTC. However, if the liquid is at its boiling temperature, then it will boil upon plunging a warmer object, forming a gas film around the specimen, leading to a small HTC and much lower cooling rates than in the former case. Liquid nitrogen (LN_2_) has been the most commonly used cryogen. It is inert, safe, relatively inexpensive, and quite readily available. However, the cooling rates attainable in cryo-TEM specimen preparation are only about 7000 K/s. In contrast, liquid ethane at its freezing point of 90 K (FEt) is a much better cryogen because its normal boiling point is 183 K. A room-temperature specimen that is plunged into FEt will not give rise to boiling and will be cooled at a rate of 100 000 K/s, as directly measured.^20^ However, FEt is a good solvent for organic solvents even at its very low temperature,^16^ thus it cannot be used for cooling most nonaqueous systems, including strong acids, which oxidize FEt violently, like many organic materials. Fortunately, most organic solvents, such as branched hydrocarbons, aromatic compounds, esters, ketones, and alcohols, and even strong acids, such as chlorosulfonic acid (CSA), can be vitrified by the relatively slow cooling rates afforded by liquid nitrogen.^15,16^ CSA is the only practical solvent for carbon nanotubes (CNTs), graphene, and boron nitride nanotubes (BNNTs), all of which we have studied by cryo-EM.^3,21^

Linear liquid hydrocarbons have a very strong tendency to crystallize, thus they cannot be vitrified in LN_2_^16^ and hence liquid systems, in which the continuous phase is made of linear hydrocarbons, cannot be imaged reliably by cryo-TEM. Replacing the linear hydrocarbon with a similar branched one can bypass this problem. In an early example, normal decane, the solvent, was replaced by Isopar M, a commercial mixture of branched hydrocarbons with physical properties close to those of decane. Small-angle neutron scattering (SANS) was used to verify that the aggregates formed in the solvent did not change as the solvent changed.^22^ In another case, for the much studied microemulsion of octane, water, and nonionic surfactant C_12_E_5_ (pentaethylene glycol monododecyl ether), normal octane was replaced by isooctane.^19^ We redid the phase diagram to establish the slightly altered phase boundaries and successfully imaged the system at different compositions and temperatures by a combination of cryo-TEM and cryo-SEM (see below).

The situation is different with cryo-SEM specimens as they are typically much larger than those of cryo-TEM, and thus their surface area-to-volume ratio is much smaller, which considerably reduces the cooling rate reached by plunging the specimen into a cryogen, even into FEt. To overcome this problem, we reduce the nucleation and growth of crystals during the thermal fixation of these specimens by cooling while the specimen is subjected to very high LN_2_ pressure, on the order of 210 MPa. This so-called high-pressure freezing (HPF) method leads to very good preservation of cryo-SEM specimen nanostructure.^23^ HPF may also be used for specimen preparation of a nonaqueous system because the high-pressure cryogen is the inert LN_2_.

### Controlled Environment

The other major challenge of cryo-EM is preserving in the specimen the nanostructure of the original sample at a specified concentration and temperature. That was achieved for the first time by Bellare et al.,^1^ who designed and built the controlled environment vitrification system (CEVS), which allows control of the temperature and the saturation of the atmosphere around the specimen during its preparation, thus keeping its desired temperature fixed and preventing changes to its concentration by either evaporation from or condensation on the prepared specimen. That basic idea has led to several commercial automated systems,^24,25^ which are easier to use by less experienced users but offer less flexibility in specimen preparation, as described below. To saturate the atmosphere in the CEVS or one of its clones, one should use the liquid of which the specimen is made. For most biological systems, pure water will do. In the case of nonaqueous systems, a liquid of the same composition of the sample (e.g., a microemulsion) should be used for reliable cryo-specimen preparation.^19^ That cannot be done with the currently available commercial systems. In the case of specimen preparation of solutions in superacids, such as CSA, one needs to work in a completely dry atmosphere because water interacts strongly with CSA, forming sulfuric and hydrochloric acids. Evaporation is not a problem because the vapor pressure of the acid is very low at room temperature. To keep the atmosphere around the specimen dry, we flush the CEVS continuously with dry air or dry nitrogen.^3^

Some time ago, we extended the concept of the CEVS to the preparation of cryo-SEM in a controlled atmosphere.^2^ The methodology allows the user to assemble the cryo-SEM specimen in the CEVS chamber and load it into specially designed tweezers and then plunge it into the cryogen. The methodology can be applied for either aqueous or nonaqueous systems’ cryo-SEM preparation.^18^ When preparing cryo-SEM specimens of acid-based specimens, the CEVS is inserted into a collapsible glovebox and flushed with dry nitrogen to minimize contact between the acid and water vapor.^3^

### Blotting

Cryo-TEM specimens are prepared by applying a small drop of the liquid, 3 to 4 μL, onto a TEM copper grid covered by a perforated film, made either of carbon-coated polymer or of silicon, with a range of micrometer-sized openings in the former or with uniform holes in the latter. A critical step is making this drop into a thin (less than 300 nm) liquid film spanning the holes in the support film. That is achieved by blotting most of the liquid away by touching absorbing paper to the drop. In the automatic commercial systems, that is done by a mechanism pressing one or two pieces of filter paper to one or two sides of the grid. The time of contact and the number of contacts can be set by the operator. This mode of blotting is efficient for low-viscosity aqueous systems.

In the original CEVS, blotting is performed manually by a piece of filter paper mounted on a metal strip, manipulated from outside the CEVS chamber through a rubber septum. In the case of specimens in CSA or other “superacids”, which are very strong oxidants, the normal cellulose paper has to be replaced by fiber-glass paper that resists oxidation by the acid. One can use different modes of blotting according to the rheological properties of the drop on the grid. Touching the paper to one or two sides of the grid is an effective option, which can be repeated several times. Touching the paper to the bottom of the grid is a very good option for very low-viscosity organic solvents. Spreading the liquid over the grid is most useful for very viscous shear-thinning liquids (many complex liquids are indeed shear-thinning) that cannot be made into thin films in any other blotting method. This produces a very high shear rate, typically larger than 10^4^ s^–1^, which reduces the liquid viscosity temporarily, allowing the formation of thin films over the substrate holes. This can be applied to all types of liquids.

One has to bear in mind that high shear rates may lead to flow-induced transient aggregates, as, for example, changing a threadlike micellar solution to a lamellar one,^26^ or vice versa.^27^ Also, the alignment of slender aggregates is sometimes observed.^28^ Most of these phenomena have short relaxation times of just a few seconds. Thus, after blotting, the specimen may be allowed to relax in the controlled environment of the CEVS, typically for 30 to 60 s, before it is plunged into the cryogen.^26^ Another effect of flow during specimen preparation is the alignment of slender objects along flow lines, forming well-ordered domains which do not exist in the bulk sample.^28^ Also, because the liquid films spanning the holes in the support file are usually biconcave, larger objects move to thicker areas near the edge of the hole, quite often leading to size-fractionation in the hole area.^28^

## Electron Beam Radiation Damage

Any microscopy depends on the interaction of the beam, or another type of probe, with the specimen. Some of these interactions are essential for recording the micrograph (i.e., elastic scattering in TEM or the backscattering of electrons in the SEM). Others are deleterious, such as the ionization of specimen atoms and the breaking of chemical bonds,^9^ which lead to major nanostructural changes in the specimen. We call the latter electron beam radiation damage (EBRD). This is especially severe in cryo-specimens and often much more so in nonaqueous specimens.

In most cases, cryo-EM specimens are made of organic molecules dispersed in water or in organic solvents. They are examined at an acceleration voltage on the order of 1 kV in cryo-SEM (see below) and at 120–200 kV in cryo-TEM, thus the major mechanism of EBRD is the ionization of atoms (which also produces secondary electrons), the formation of free radicals, and the breaking of chemical bonds. Water is a major source of free radical formation under the electron beam, giving rise to free-radical chain reactions at the water–organic material interface. Counterintuitively, the rate of damage is faster at lower acceleration voltage in the range used for EM because the probability of an ionization event increases at lower electron energies in the given range.^29^ In aqueous systems, the damage manifests itself most clearly by mass loss at electron exposures of around 20 e^–^/Å^2^. Specimen crystallinity, when present, is most sensitive to the electron beam and is damaged even at very small electron exposures of just a few e^–^/Å^2^.^29^ Most cryo-TEM work is performed with perforated carbon films, which are actually carbon-coated perforated polymer films. In the presence of vitrified water, they show the first signs of mass loss after about 20 e^–^/Å^2^ exposure, at an exposure rate of about 1 to 10 e^–^/Å^2^/s, thus acting as a built-in rough indicator of the electron exposure.

Very few studies of EBRD in the cryo-EM of organic liquids have been published. These have shown quite unexpected phenomena. In pure vitrified organic solvents, the damage is mostly mass loss, seen as small holes that increase in size with accumulated electron exposure. When organic aggregates are embedded in a vitrified organic liquid, some unexpected changes with electron exposure are imaged. These can lead to contrast reversal^30^ or an increase in image contrast.^31^ If water is also present, then radiation damage is faster and is expressed also in increasing mass loss under the beam.^32^

EBRD of vitrified specimens of CNTs, graphene, or BNNTs in CSA is similar to that of vitrified aqueous specimens under the electron beam; namely, radiation damage starts at the interface of the vitrified acid with nanotubes or graphene sheets.^3,33^ The mechanism is not quite clear but may be related to free radical formation on the carbon or the boron nitride acid-modified surface.

To reduce EBRD, the operator should minimize the electron exposure of the specimen. That is true for either aqueous or nonaqueous systems in either cryo-TEM or cryo-SEM. First, one has to keep in mind that for the same brightness the electron exposure that is needed increases with the square of magnification. Thus, the minimum magnification needed for the required resolution should be used. Modern TEMs have low-dose imaging protocols available in their software. Those allow the operator to adjust all of the instrument parameters on one area and move automatically to another, the target area, to be imaged without earlier exposure. A similar approach is used in cryo-SEM, although not assisted by software yet.

While EBRD is a major deleterious effect of the electron beam, it can be used, if applied carefully, to enhance the contrast by selectively affecting one type of material relative to another. We showed that effect in cryo-TEM for aqueous systems, as, for example, to differentiate between two different polymer latexes suspended in water, each undergoing EBRD at a different rate.^9^ This same approach can be used to enhance the contrast between CNTs or graphene in vitrified CSA. Mass loss at the interface of the suspended material and the acid, while the specimen is exposed to the beam, makes them clearly visible.^3^ In other nonaqueous systems, such selective etching can lead to the identification of different materials and provide evidence of the presence of water in certain domains in the system.^32^

Because in cryo-SEM the required resolution is often much lower than that of cryo-TEM, we do not see fine details of EBRD. However, quite often we do see mass loss after some electron exposure. Because in the SEM we have the option of scanning only part of the final micrograph frame, before we record the picture, we are able to compare high- and low-electron exposure areas of the micrograph quite easily. Selective beam etching is useful here, too, for example, as in removing some of the CSA to expose well-ordered CNTs in a lyotropic liquid-crystalline phase.^3^

## Imaging

Cryo-EM has benefitted much from the progress in electron microscopy in recent years. In TEM, we have seen progress made in the technology of field emission guns (FEGs), electron optics, and electron cameras. Another major advancement has been the introduction of the Volta phase plate for image contrast enhancement. In SEM, we have also witnessed the introduction of efficient easy-to-use FEGs and new imaging system design such as the Gemini column introduced by Zeiss or the specimen electron retardation landing potential introduced by FEI (now Thermo Fisher). Cryo-holders and cryo-stages to keep the cryo-specimens at the needed temperature (−150 °C or lower) have become reliable and so are the cryo-pumps (large metal surfaces close to the specimen, kept at cryogenic temperature to trap residual molecules in the EM vacuum), which have also become quite efficient. Modern EMs are now equipped with oil-free vacuum systems that minimize the number of condensable molecules in the microscope column. Below we describe how we take advantage of this progress on cryo-TEM and cryo-SEM of nonaqueous liquid systems.

### Cryo-TEM

Many of the specimens one images in cryo-TEM, in general, and in cryo-TEM of nonaqueous systems, in particular, have inherent low image contrast. In some cases, as described above, we can enhance the contrast by selectively controlled etching by the electron beam. However, this approach is not desirable in most cases. Another common way to enhance image contrast is by defocusing the TEM objective lens, thus converting image phase differences to amplitude differences. That, however, leads to a loss of resolution. In modern TEMs, the image contrast may be enhanced by the so-called Volta phase plate,^34^ analogous to the Zernike glass phase plate used in phase-contrast light microscopy.^35^ The Volta phase plate is made of a thin polymer film inserted into the electron path instead of the objective aperture, and when charged by the electron beam, and as in the Zernike case, it gives a phase shift of π/2, which leads to the conversion of image phase differences to amplitude differences. That allows for in-focus, or slight underfocus, imaging without a loss of resolution and with good contrast.

Most complex liquid specimens are very sensitive to EBRD. To minimize beam damage, we apply low-dose imaging, as described above. The number of electrons used to record the image can be minimized by the new generation of detectors, namely, direct-imaging cameras that record each electron with no intermediate scintillation step. With such cameras, it is possible to record images with little noise and an excellent grey-scale range with as little as 2 e^–^/Å^2^.

### Cryo-SEM

The current technology of SEM has allowed us to use the instrument with cryo-specimens in a way not possible before. The current FEG technology makes imaging at low acceleration voltage (LAV) quite routine. Because the brightness of the electron source decreases with acceleration voltage, only an FEG makes it possible to operate an SEM below 5 kV with an acceptable signal-to-noise ratio. LAV is needed to minimize the volume of interaction of the beam in the specimen, a prerequisite for high-resolution SEM (HR-SEM). Thus, all HR-SEMs must be FEG-equipped. Also, for most materials at some specific LAV on the order of 1 kV, the number of electrons leaving the specimen equals the number of electrons impinging on it, thus even nonconductive specimens are not electrically charged by the beam,^36^ even without a conductive coating, which is often applied on top of such specimens. This saves a step in cryo-specimen preparation and prevents the loss of resolution due to the coating grains. It is important to emphasize here that micrograph contrast depends on the exact voltage used, around the point of neutrality, and could be increased, minimized, or reversed by relatively small changes in the acceleration voltage, especially between conductive and nonconductive domains, such as in the CNT nematic phase in CSA.^4^ At LAV, the physics of the electron–matter interaction is quite different than at higher voltage,^37^ which has important practical implications for imaging by the HR-SEM, including cryo-specimens, a fact that is not clear to and not taken advantage of by many SEM practitioners.

The current technology of electron detectors is also most helpful. In most modern HR-SEMs, there are two detectors for secondary electron imaging (SEI) and two for backscattered electrons (BEI), one of each outside the column and one of each inside the column. The latter two are used for high-resolution imaging; the secondary electron detector outside the column is useful for the topographical contrast of fractured specimens. The in-the-column BEI detector is used to obtain elemental contrast, even between elements very close on the periodic table of the elements, such as carbon and oxygen (e.g., the contrast between oil and water;^4^ see below). Signals from those detectors can be combined, if necessary. They can be augmented by elemental mapping based on the specific energy of the X-rays emitted from the different elements (energy-dispersive spectroscopy, EDS).^38^

## Some Recent Results

### Materials

In our work, we used two types of carbon nanotubes (CNTs): as-received CCNI 1109 CNTs purchased from Carbon Nanotechnologies, Inc. (Houston, TX) and Meijo EC 1.5p CNTs obtained from Meijo Nano Carbon Co. Ltd. (Nagoya, JP). These were purified at Rice University by H_2_O_2_ liquid-phase oxidation, followed by HCl treatment.^39^ Boron nitride nanotubes (BNNTs) in this study were synthesized at BNNT LLC (Newport News, VA). The synthesized BNNTs were purified to remove elemental boron (batch designated SP10R) and further purified by a high-temperature steam treatment to remove h-BN (batch designated SP10RX).^40^ For the spontaneous dissolution of CNTs and BNNTs in superacid, without any treatment or sonication,^41,42^ we used ACS-certified chlorosulfonic acid (CSA) of 99% purity, purchased from Sigma-Aldrich (St. Louis, MO).

We also used a commercial surfactant-stabilized water-in-silicone oil emulsion, courtesy of the L’Oréal Research & Innovation Department, Paris, France

### Cryo-TEM

Cryo-specimens of acid-based solutions and dispersions were prepared in a home-built CEVS in a dry-nitrogen atmosphere, as described elsewhere.^1^ The cryo-specimens were vitrified in liquid nitrogen and loaded under controlled condition into a Gatan 626 cryo-holder and kept in the TEM at −180 °C. Imaging was performed with an FEG-equipped FEI (now Thermo Fisher Scientific) Talos 200 C TEM, operated at 200 kV, using the low-dose software of the TEM. Contrast was enhanced by the Volta phase plate with a small objective lens underfocus of 200 to 500 nm. Images were recorded by a Falcon III direct-imaging camera using the TIA software package.

**Figure 1:**
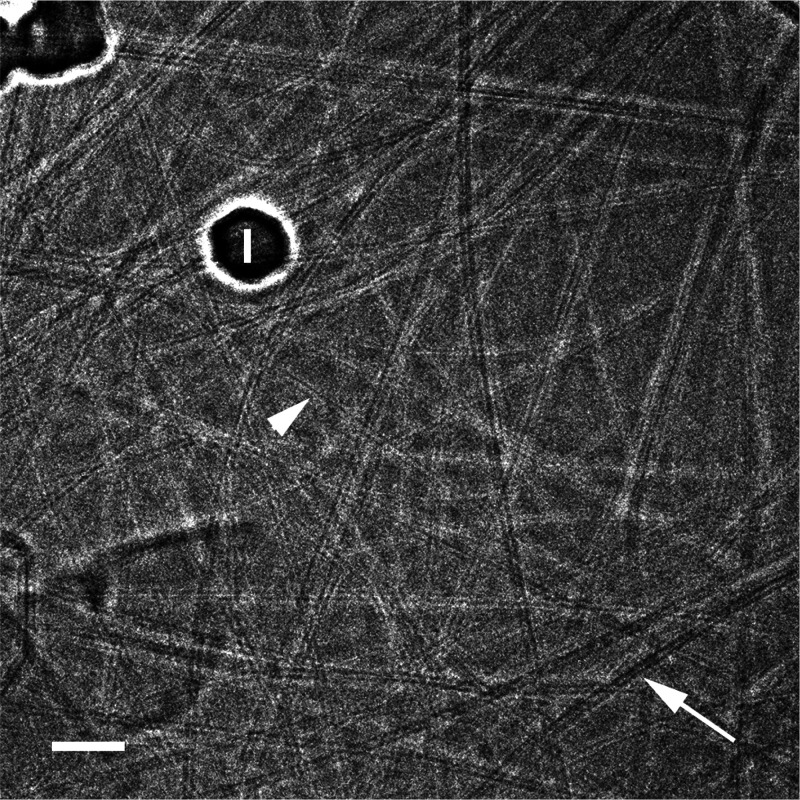
Vitrified specimen of 30 ppm CCNI 1109 CNTs in CSA. The image was taken with the Volta phase plate at low electron exposure. Note the filled (arrowhead) and empty CNTs (arrow). “I” denotes an ice crystal, deposited on the specimen during transfer into the TEM. The scale bar corresponds to 50 nm.

Figure 1 shows a vitrified CNTs in CSA dispersion. The image shows the fine details of the 4.1 nm outer-diameter single-walled CNTs, including their walls. The background is vitrified CSA. Similar optical density inside and outside some CNTs (arrowhead) indicates that those CNTs are acid-filled. Others (arrow) are empty, giving better contrast with the walls.

**Figure 2:**
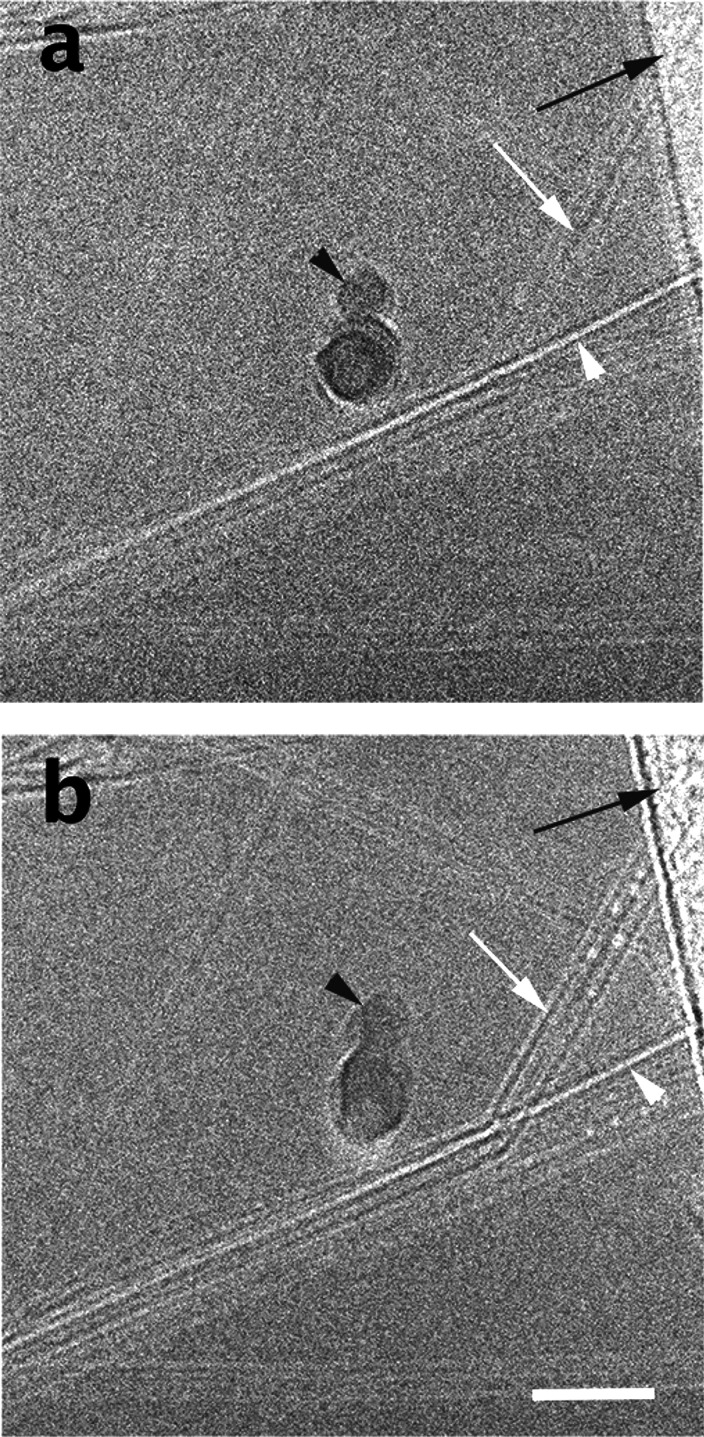
Vitrified specimen of 0.4 wt % SP10_R BNNTs in CSA. (a) Low-exposure image at about 10 e^–^/Å^2^. (b) Same area at ∼40 e^–^/Å^2^. Note the changes due to EBRD, including improved image contrast. Black arrows point to the support film, white arrowheads point to an empty BNNT, and black arrowheads point to an ice crystallite. The scale bar corresponds to 50 nm.

In Figure 2, we show a vitrified specimen of 0.4% SP10_R BNNTs in CSA at low-electron exposure (Figure 2a) and at a higher exposure (Figure 2b). In the former, the image contrast is low. However, we see quite well the one empty nanotube indicated by a white arrowhead. The acid-filled nanotubes are barely visible. The black arrowhead points to a crystalline ice particle that landed on the specimen during transfer. The black arrow points to the perforated carbon film on which the specimen was prepared. Following additional exposure to the beam, the filled BNNTs become visible (white arrow). We also notice etching of the vitrified acid, seen as lighter holes. The ice particle is also changed by irradiation due to the additional free radicals generated in the ice. Considerable damage is also seen on the support film (black arrow in Figure 2b).

Micrographs such as those seen in Figure 2 allow the assessment of the quality of the BNNTs, their purity, and their dissolution in the acid, which are all important for achieving the final goal of wet-spinning of high-quality fibers from BNNTs, similarly to the wet spinning of high-quality fibers from CNTs.^43^

**Figure 3:**
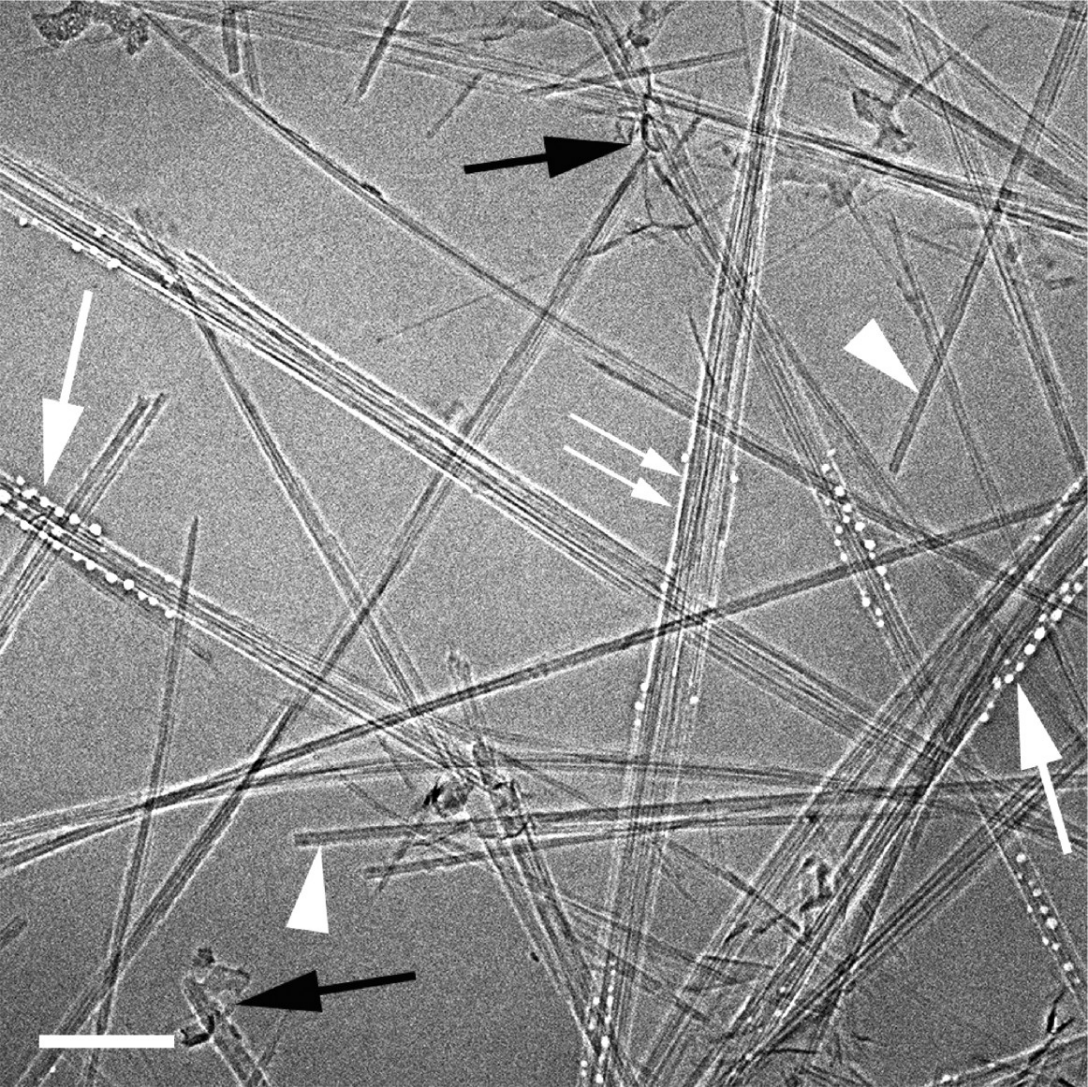
Vitrified specimen of 0.4 wt % SP10_RX BNNTs in CSA showing bundles of BNNTs and individual ones (arrowheads). Black arrows indicate boron nitride debris. White arrows point to the area where EBRD has started. The double arrow shows a line of EBRD at the BNNTs–acid interface. The bar corresponds to 100 nm.

Figure 3 is a cryo-TEM image of a vitrified specimen of 0.4% SP10_RX BNNTs in CSA. It shows bundles of BNNTs along with individual ones (arrowheads). Black arrows indicate boron nitride debris, which is deleterious for fiber spinning. White arrows point to the area where EBRD has started, appearing as round holes, where acid was etched away, quite similarly to what is observed in aqueous cryo-TEM specimens.^44−46^ Why the process starts at certain specific sites is still unknown. Double-arrow shows a line of EBRD at the BNNTs-acid interface.

**Figure 4:**
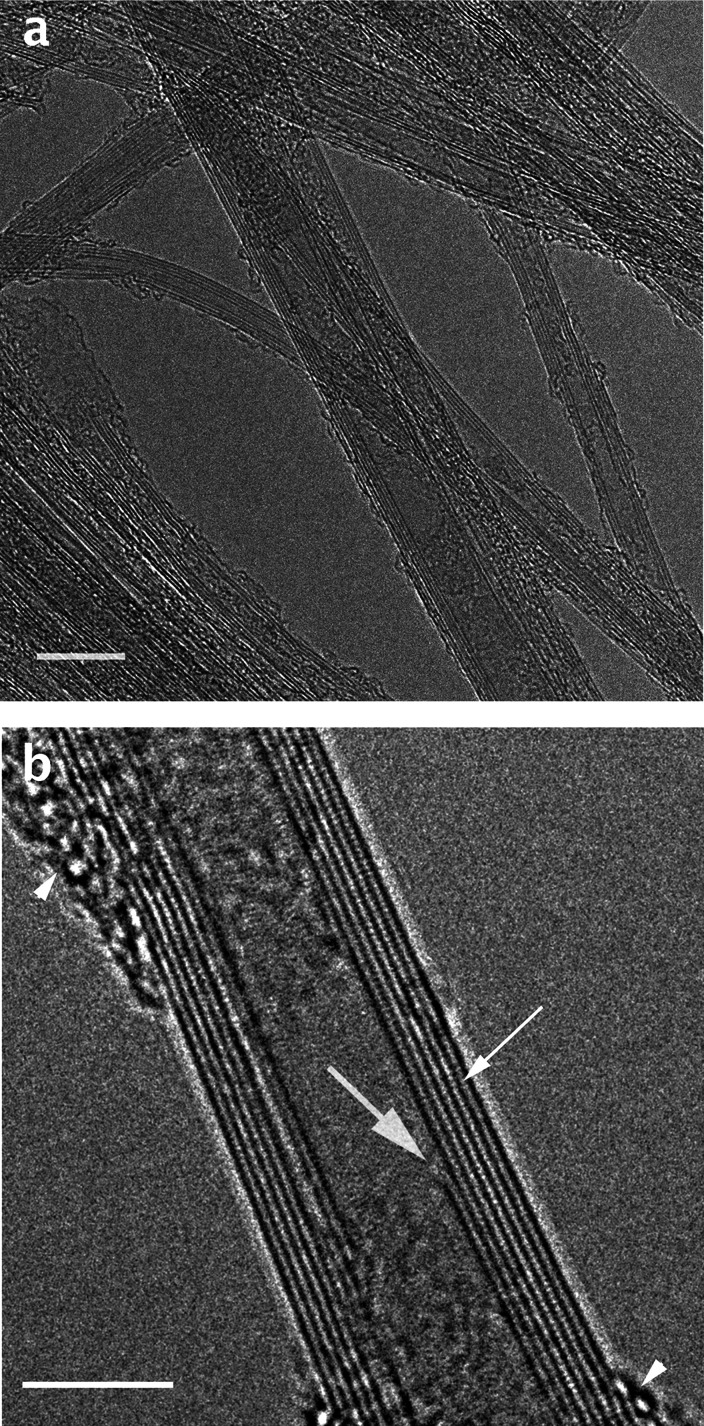
HR-TEM room-temperature images of BNNTs: (a) a relatively large field of view, with several BNNTs, partially overlapping; (b) a very high-resolution image of a multiwalled BNNT. The fine arrow points to a B–N pairs, making up the walls; a thick arrow indicates discontinuity of the inner wall; arrowheads point to debris covering part of this tube section. Bars correspond to (a) 10 nm and (b) 5 nm.

Another use of cryo-TEM specimens is as intermediate step in preparing specimens for HR-TEM, overcoming the difficulty of depositing individual or small bundles of nanotubes (CNTs or BNNTs) on a perforated carbon film. The first step is the preparation of a cryo-TEM vitrified specimen of the nanotubes in CSA, as described above. Then, the cryo-specimen is quenched in room-temperature distilled water, rinsed several times in water, and dried. The water disintegrates the acid, leaving behind well-dispersed nanotubes supported over the holes in the perforated carbon film. This procedure gives very clean, preparation-artifact-free specimens. Figure 4 shows high-resolution images of room-temperature BNNTs specimens prepared by this protocol. Figure 4a shows a relatively large field of view with many BNNTs. It resolves clearly the number of concentric tubes in the BNNTs and the structural defects in them. Figure 4b is a very high resolution image of a multiwalled BNNT. The walls appear as lined-up spheres (fine arrow), each of which is a B–N pair. Note the discontinuity of the inner wall (thick arrow). The arrowheads point to debris covering part of this tube section.

### Cryo-SEM

We used a Zeiss Ultra Plus HR-SEM equipped with a Leica (formerly Bal-Tec) VCT100 cryo-holder (and transfer system) maintained at −145 °C. We used the Everhart Thornley (ET) secondary detector to record the specimen fracture surface topography. For higher-resolution SEI, we used the in-the-column SE detector (InLens detector in the Zeiss terminology). For BSI, we used the in-the-column detector (EsB detector in the Zeiss terminology), which gives good elemental contrast. In all SEM figure captions, we give the acceleration voltage (AV) and the work distance (WD), namely, the distance between the specimen and the lowest lens of the SEM. WD affects the micrograph depth of field and resolution.

**Figure 5:**
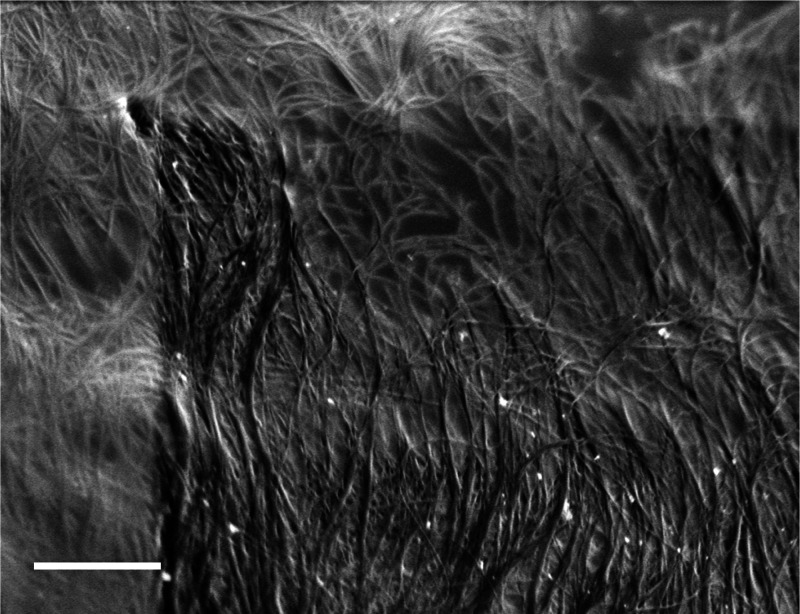
Cryo-SEM SEI micrograph of 3 wt % 1.5 Meijo CNTs in CSA. The darker area was irradiated longer than the lighter area, exposing the CNTs after etching away some of the CSA, showing clearly bundles of CNTs, forming a nematic liquid-crystalline phase. The bright spots are nanoparticles of the iron catalyst exposed by the etching. AV = 1.4 kV, WD = 2.8 mm, and the scale bar corresponds to 500 nm.

Figure 5 is a cryo-SEM SEI micrograph of 3% CNTs (of the 1.5 Meijo type) in CSA. At this rather high CNT concentration, domains of a nematic lyotropic liquid-crystalline phase are formed. Those are essential for wet-spinning of CNT high-quality fibers. The darker area in the micrograph was irradiated longer than the lighter area, exposing the CNTs after etching away some of the CSA by the electron beam, which disintegrates the acid but leaves the CNTs intact.^3^ The loss of acid makes the bundles of CNTs, forming a nematic liquid-crystalline phase, stand out clearly. The change in contrast is due to the loss of acid relative to the exposed highly conductive CNTs.^3^

**Figure 6:**
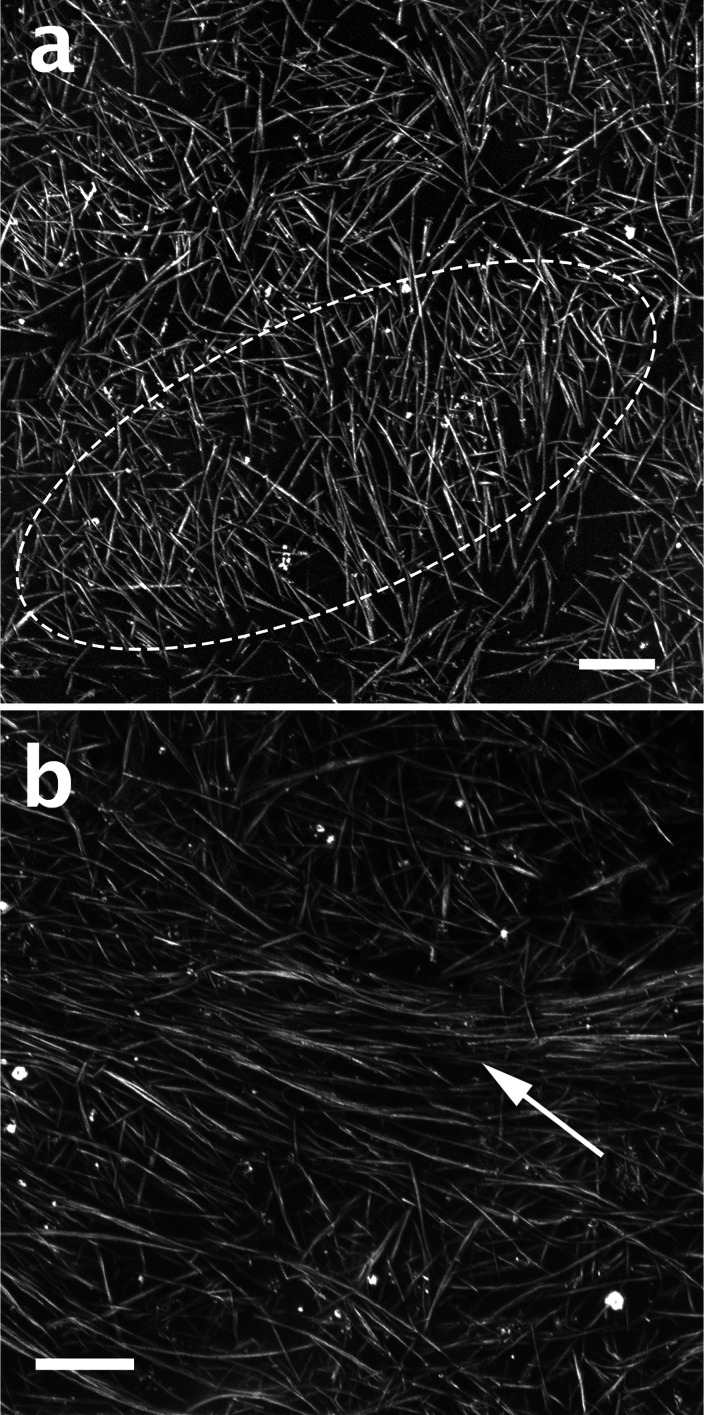
Cryo-SEM micrographs of 0.8 wt % SP10RX BNNTs: (a) A short-range ellipsoidal nematic domain, marked by dashed lines; note BNNT bundles ordered in parallel to the minor axis of the ellipse (b) A nematic liquid-crystalline domain (arrow) surrounded by the isotropic CSA solution. The bright spots here are most probably h-BN nanoparticles. AV = 0.6 kV. WD = 3.6 mm (a) and 3.7 mm (b). Scale bars correspond to 1 μm.

Cryo-SEM micrographs of 0.8% SP10RX BNNTs in CSA solution are presented in Figure 6. At this concentration, the BNNTS begin to form ordered domains. Figure 6a shows a short-range ellipsoidal nematic domain, with BNNT bundles ordered in parallel to the minor axis. Figure 6b shows a nematic liquid-crystalline domain surrounded by isotropic solution. This is an ongoing project.

**Figure 7:**
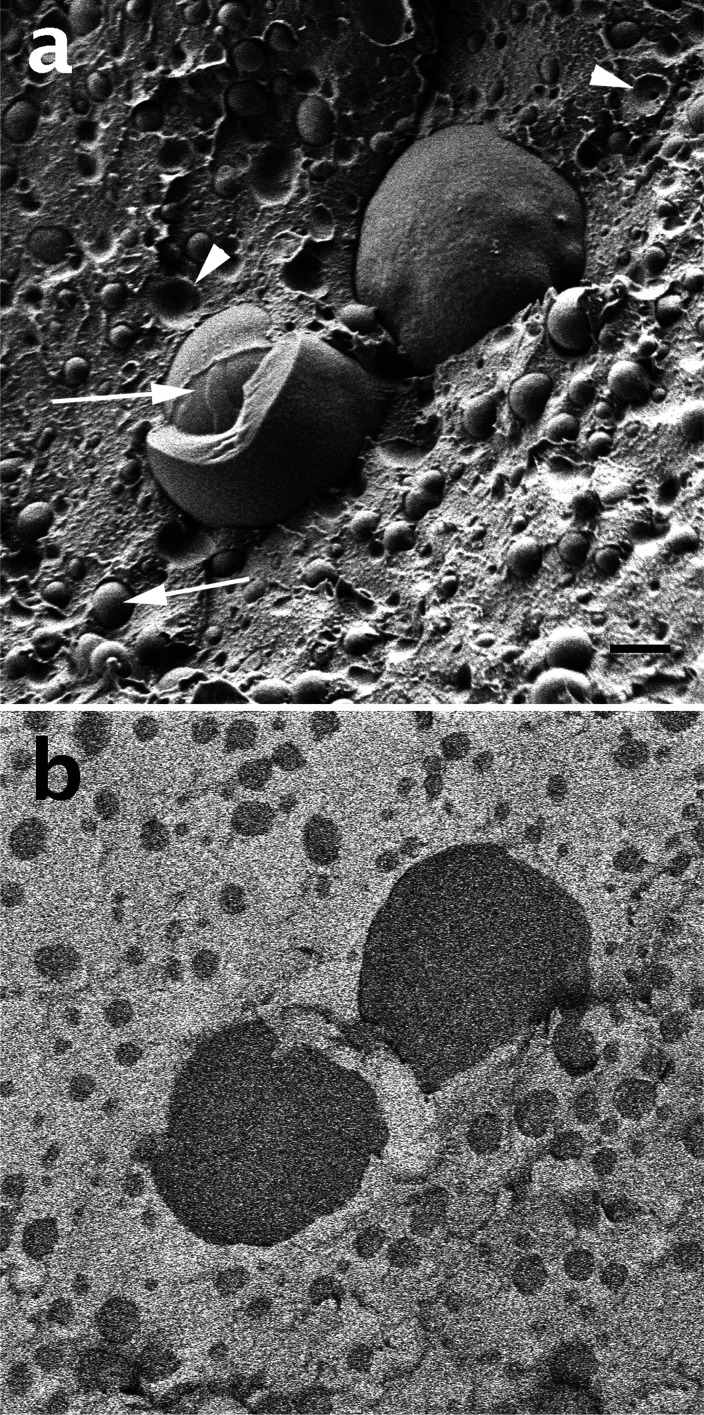
Cryo-SEM micrographs of a commercial surfactant-stabilized water-in-silicone oil emulsion: (a) Secondary electron micrograph; the arrow points to water globules, and arrowheads point to craters left by droplets plucked during specimen fracture. (b) The same area as in panel a, imaged by backscattered electrons. The lighter area represents the silicone oil, and the darker area represents the water. AV = 1 kV, WD = 3.8 mm, and scale bar = 1 mm.

Figure 7 demonstrates the application of different imaging detectors in cryo-SEM. In this example, we imaged a commercial surfactant-stabilized water/silicone oil emulsion. On the basis of the cryo-SEM results, it became clear that in this case the formulation was an oil-in-water emulsion. Figure 7a shows the SEI micrograph. We see globules of the dispersed phase (arrows) embedded in the continuous phase along with craters from which the globules had been plucked out during the fracture step of the cryo-SEM specimen preparation. While this shows that indeed we have here an emulsion and it gives us information about the droplet size distribution, we do not know which type of emulsion it is. Figure 7b is the BEI micrograph of the same area as in Figure 7a. The continuous phase appears here to be much brighter than the dispersed phase, indicating that it is the silicone oil, composed of silicon and oxygen, while the water is rich only in oxygen. Because silicon is a much heavier atom than oxygen, it gives more backscattered electrons per every electron of the beam, thus the silicone oil regions appears lighter in the micrograph. We recently published an opposite example of an oil-in-water emulsion, where the continuous water appeared brighter than the oil phase due to more backscattered electrons produced by oxygen (water) than the oil (carbon).^4^ Note that the BEI micrograph lacks any topographical information.

## Concluding Remarks

Cryo-TEM of liquid aqueous biological and synthetic material systems has become almost routine in many academic and industrial laboratories. Cryo-SEM of such systems has also established itself as a useful methodology, although on a more limited scale. Work on nonaqueous liquid systems or, more specifically, complex liquids, in which the continuous phase is nonaqueous, has been lagging far behind. In this Account, we have described the difficulties associated with cryo-TEM and cryo-SEM of nonaqueous specimens. We have shown the specific challenges posed by those systems in specimen preparation and in the interaction of the beam with the specimen, including imaging and electron-beam radiation damage. We have shown how a good understanding of the physics of specimen preparation and imaging allows us to extend cryo-EM to reliable high-resolution imaging of nonaqueous complex liquids, thus broadening the range of applications of the methodology. We have demonstrated the success of the process of adaptation of the methodologies by several examples of cryo-TEM and cryo-SEM. We hope that this will lead to a more widely spread application of these methodologies.
